# Overlap or breakthrough? exploration of the academic buoyancy structure in Chinese EFL learners

**DOI:** 10.1371/journal.pone.0318347

**Published:** 2025-01-31

**Authors:** Tao Zeng, Ke Zhong, Wanting Wang

**Affiliations:** 1 College of Foreign Languages, Hunan University, Changsha, China; 2 Hunan Provincial Research Center for Language and Cognition, Changsha, China; 3 School of Foreign languages, Changsha Medical University, Changsha, China; Ahvaz Jundishapur University: Ahvaz Jondishapour University of Medical Sciences, ISLAMIC REPUBLIC OF IRAN

## Abstract

Academic buoyancy, recognized as a key component of positive personality traits in learning, has garnered significant attention. However, most research on buoyancy is limited to general educational contexts, lacking a comprehensive theoretical framework that poses challenges in evaluating buoyancy’s impact on learners within the domain of second language acquisition (SLA). This study seeks to bridge this gap by investigating academic buoyancy within the realm of foreign language learning, specifically among university students in mainland China studying English as a foreign language (EFL). The study comprises two phases, with a total of 632 EFL participants. In the initial phase, a 32-item scale was tailored to the present study, drawing on scales from previous buoyancy-related research. Item analysis and exploratory factor analysis were subsequently conducted with a sample of 209 students. The phase retained 21 measurements and identified three main components of buoyancy: sustainability (the ability to persist despite difficulties), goal-orientedness (the focus on achieving specific learning objectives), and controllability (the perception of managing learning challenges). In the second phase, the refined questionnaire was administered to an additional 423 students, and the data underwent confirmatory factor analysis. A reliable 12-item scale was obtained that accurately reflects the identified components of academic buoyancy. By elucidating the structure of buoyancy, this study offers valuable empirical insights that can guide pedagogical strategies and strengthen learner buoyancy in language learning contexts. The findings contribute to the broader discourse on positive personality traits in education, highlighting the importance of fostering buoyancy in language learners to support their academic success.

## Introduction

Positive Psychology (PP) explores how people utilize their strengths to cope with challenges and enhance these abilities [1–4]. It focuses on three pillars: positive experiences, personality traits, and institutions [5], all of which are crucial for second language acquisition (SLA). Research has investigated PP’s influence on SLA, focusing on factors such as happiness, perseverance, caring teaching, engagement, emotional regulation, resilience, and well-being [4]. Yet, areas like optimism, connectedness, commitment, academic buoyancy, immunity, and mindfulness need further exploration [1].

Academic buoyancy is a cognate structure of academic resilience but differs from resilience by focusing on how students handle minor daily academic challenges, while resilience deals with overcoming significant difficulties [6,7]. Unlike coping, which is about specific strategies for addressing problems, and immunity, which relates to biological defense mechanisms, academic buoyancy is concerned with everyday setbacks [8,9]. Recently, international scholars have increasingly studied academic buoyancy, exploring its definitions, dimensions, influential factors, structure, and measurement in the context of second language education [e.g., 6].

However, gaps remain in thoroughly exploring the applicability of its foundational notions and structure in the field of second language acquisition (SLA) [10]. Unlike other disciplines, SLA involves not only knowledge acquisition but also the integration of diverse cultural elements, which leads to more complex academic challenges for second language (L2) learners. Therefore, recognizing the psychological resilience and adaptive capacities encapsulated by the “structure of buoyancy” among these L2 learners is increasingly acknowledged as crucial for advancing understanding of their attitudes, confidence, and self-efficacy in the language learning process and improving educational outcomes [11–14].

This study focuses on exploring the structure of academic buoyancy in Chinese students learning English as a foreign language (EFL) and aims to investigate the relationship between the psychological attributes associated with academic buoyancy within the context of foreign language learning and those identified in previous research. In contrast to earlier research, this study thoroughly considers the behavioral and emotional components in addition to accounting for the psychological factors that have been linked to the buoyancy. The primary goal is to enhance the understanding of the buoyancy structure among Chinese L2 learners. Additionally, to ensure the stability and reliability of this structure, this study employed two rounds of data collection with separate datasets for distinct analytical purposes and utilized multilayer statistical analyses to determine whether there is an overlap or any innovation between these attributes. Prior studies on buoyancy structure noticeably lack this kind of experimental procedure. The results of this study are anticipated to offer valuable insights and serve as a reference for future research on academic buoyancy in the domain of SLA.

## Literature review

### Positive psychology in SLA

Unlike the traditional deficit-oriented approach of previous psychology, PP emphasizes not just addressing weaknesses but also identifying and enhancing human strengths [15]. It aims to help individuals achieve success and realize their potential by creating and developing these strengths [5]. Its goal is to help individuals achieve success and reach their potential by cultivating these strengths [4].

Since the introduction of PP to SLA, MacIntyre has highlighted several key contributions relevant to foreign language learning [5]. First, PP emphasizes shifting from negative to positive emotions, showing the potential benefits of emotions in L2 contexts. Second, the character strength model in PP, which includes the six elements of justice, wisdom, transcendence, courage, temperance, and humanity [16], is believed to improve L2 learning and teaching. Additionally, Oxford’s nine-component model related to well-being, including emotion, meaning, perseverance, and autonomy, and others, offering new avenues for empirical research in L2 education [17]. To promote the performance and well-being of foreign language learners, the PP movement has encouraged numerous L2 researchers to investigate the positive emotions and personality traits of both language teachers and learners [e.g., 3,18]. However, among these traits, academic buoyancy, which can directly reflect learners’ ability to cope with dilemmas on a daily basis, had been neglected.

This concept is especially relevant to second language acquisition, given that teaching and learning languages are widely recognized as some of the most demanding and emotionally charged tasks globally [19].

### Structures of academic buoyancy

Academic buoyancy representing the conceptual framework that encapsulates the day-to-day academic resilience within the paradigm of positive psychology [10]. It is closely linked to students’ ability in overcoming academic setbacks and challenges prevalent in educational settings, including subpar grades, competing deadlines, heavy test pressure, and difficult assignments [6]. Key principles underpinning academic buoyancy encompass building on strengths and encouraging students to take a proactive rather than reactive approach to these setbacks and challenges [7].

Numerous studies have investigated indicators measuring academic buoyancy and the positive impact of academic buoyancy on various educational and psychological outcomes. Research has shown that performances of buoyancy typically map to self-efficacy, low anxiety, controllability, strategic planning, and sustainability [20]. It is also marked by diligence [21], looking to the future [22], and regularity adaptation [23,24] due to differences in cultural backgrounds and learning environments. The researchers further found that buoyancy significantly and positively predicted enjoyment of school, class participation, and general self-esteem [20,25,26], personal best efforts [27], general academic achievement [28,29], and psychological or school-related well-being [30,31]. In other words, students demonstrating higher levels of buoyancy (e.g., sustainability or adaptability) tend to achieve superior academic performance and attain more favorable outcomes. Overall, academic buoyancy has made significant progress within educational contexts globally (see 6 and the like).

The structure of buoyancy was initially explained by Martin and Marsh’s ‘5C’ model [20], a motivational dimension that includes control, confidence (high self-efficacy), coordination (high planning), composure (low anxiety), and commitment (high persistence). They found that buoyant students typically possess qualities of confidence and persistence through a longitudinal study of 1,866 high school students. These students can effectively manage their learning, set clear objectives, and exhibit lower levels of anxiety during their studies. This mindset enables them to achieve better academic performance. In light of the 5C model of buoyancy, Comerford et al. characterized the structure of academic buoyancy of Irish second year students through an ethnographic approach [21]. The results supplemented the 5C model with the feature of autonomy, while the role of anxiety was eliminated.

To enhance the effectiveness of corrective feedback for students’ learning and to support buoyancy research with empirical data, Ahmed Shafi et al. investigates the structure of academic buoyancy under specific adverse conditions during feedback assessment [22]. They employ a mixed-methods approach, combining qualitative and quantitative analysis, to extract data by thematic analysis from the feedback of 91 university students. The results indicate that buoyant students in response to evaluative feedback can be identified through five indicators, namely, “an internal locus of control,” “understanding the grade,” “being forward looking,” “being improvement focused,” and “being action orientated”. These indicators further underline the behavioral characteristics of buoyant students when dealing with potentially negative academic issues.

Meanwhile, considerable attention has been given to the behavioral and emotional outcomes associated with buoyancy. Existing research has discovered that academic buoyancy can enhance a person’s classroom engagement [18,32,33] and problem-solving ability [34]. Students with high levels of buoyancy demonstrate stronger motivation, hold more positive self-beliefs, and make more adaptive responses to setbacks [35]. Collie et al. revealed that academic buoyancy has the potential to mitigate the impact of academic anxiety on learning strategies [36], and it has a close association with feelings of enjoyment [37] and boredom [18]. It affects language learning in a way that promotes students’ emotion [38].

In addition to research on the multifaceted nature of academic buoyancy and its implications for educational practice, the measurement of buoyancy has also received a great deal of attention in previous research, as detailed in the following section.

### Measurements of academic buoyancy

There are three scales that have been used to measure buoyancy in past research, which were developed in different contexts.

Martin and Marsh developed an Academic Buoyancy Scale, tested on 598 Australian students aged about 14, which includes four items related to handling academic stress and setbacks [6]. The scale, rated from 1 to 7 by students, shows strong reliability and consistency based on data collected twice in a longitudinal study (Cronbach’s *α* = .80 to .82, test-retest *r* = .67). It has proven effective in predicting academic outcomes across various countries and disciplines 10,24,25] but is limited by its narrow scope, covering only a few aspects of student buoyancy.

Comerford et al. used a mixed-methods approach to develop the Student Buoyancy Instrument (SBI) [21], which includes 39 items adapted from existing scales on self-efficacy, planfulness, anxiety, industry, and locus of control from the International Personality Item Pool (IPIP) [39]. The SBI scale, tested for reliability and validity with 581 second-grade students in Irish middle schools, showed acceptable results but lacks specific details on buoyancy-related items. Given the extensive 3,320-item IPIP, the practical value of this scale is limited.

In the field of SLA, Jahedizadeh et al. conformed a four-factor scale with 27 items to measure students’ buoyancy in language learning [23]. The factors are “sustainability,” “regularity adaptation,” “positive personal eligibility,” and “positive acceptance of academic life,” assessed using a five-point Likert scale. The scale includes statements such as handling failures well (sustainability), motivating oneself despite reluctance (regularity adaptation), having the energy for assignments (positive personal eligibility), and viewing academic situations positively (positive acceptance of academic life). However, the formation process of the four dimensions of buoyancy was not clear, and the phrasing of the fourth item appeared to be a measure of “positive personal eligibility” as well. The study did not conduct an in-depth discriminant validity analysis of the four dimensions, leading to doubts about the classification of “positive personal eligibility” and “positive acceptance of academic life” due to a lack of supporting data. This limitation hinders the feasibility of implementing intervention measures for scholars and teachers.

In summary, existing research sets the stage for understanding the diverse dimensions of academic buoyancy and their impact on educational practice and student well-being. However, research on the structure and measurement of buoyancy still lacks systematic and generalizability. Given the large number of English learners in China and the prominence of local characteristics, it would be imprudent to indiscriminately apply existing frameworks or measurement tools to assess the buoyancy levels of Chinese EFL learners. Therefore, this study aims to situate itself within the context of Chinese culture, focusing on the field of second language acquisition to explore the structural components of academic buoyancy in foreign language learning and confirm a corresponding measurement scale.

### The study

This research aims to delve into the structures of buoyancy and create a scale to measure the buoyancy of Chinese EFL learners. To achieve this goal, two phases were conducted. First, a questionnaire was compiled based on the relevant literature on buoyancy and then distributed. The collected data were submitted to item analysis and exploratory factor analysis (EFA) to explore the specific dimensions of academic buoyancy structure in foreign language learning. Subsequently, the identified dimensions and scale items were developed into a new questionnaire, which was then administered to a different sample. Confirmatory factor analysis (CFA) was performed on the recycled data to validate the conceptual and theoretical construct of buoyancy, and descriptive statistical analysis was then used to present the levels of psychological attributes within each dimension of buoyancy among the EFL learners. The specific research questions are listed as follows:

RQ 1: What is the structure of academic buoyancy among the Chinese college EFL learners?
RQ 2: How about the validity of the academic buoyancy structure?

## Methodology

### Participants

In this study, 710 students from Chinese universities were recruited through convenience sampling for the two phases. A total of 78 responses were removed from the analysis due to incomplete questionnaire or identical answers to all items. The final valid datasets comprised 632 responses. Specifically, sample 1 consists of 209 valid responses with 83 males (39.7%) and 126 females (60.3%). Sample 2 involves 423 students, including 206 males (48.7%) and 217 females (51.3%). They were non-English majors, such as Departments of Humanities, Natural sciences, Arts and others, with an average age of 20 years old. All participants were enrolled in a mandatory College English course and were EFL learners. None of them had experience abroad. The detailed demographic information of the participants were listed as Table 1.

**Table 1:** The demographic information of the participants.

Datasets			*n*	%
Sample 1	Gender	Male	83	39.71
		Female	126	60.29
		total	209	100.00
	Age	18	25	11.96
		19	77	36.84
		20	68	32.54
		21	19	9.09
		22	20	9.57
		total	209	100.00
Sample 2	Gender	Male	206	48.70
		Female	217	51.30
		total	423	100.00
	Age	18	40	9.46
		19	218	51.54
		20	131	30.97
		21	27	6.38
		22	7	1.65
		total	423	100.00

Note. N = 632.

### Instruments

A two-section survey was developed for this study. The first section included several questions for collecting participants’ individual information, including their gender, age, college, major, English level, and others. The second section comprised a 32-item Likert-type scale assessing participants’ buoyancy, rated on a scale from 1 (strongly disagree) to 5 (strongly agree), formulated based on a comprehensive review of existing constructs related to buoyancy (see Appendix A). The selection of scales is grounded in three primary viewpoints: firstly, drawing from established buoyancy structures like self-efficacy, perseverance, control, and planning evident in prior research [40–43]. Secondly, encompassing behavioral attributes typified by buoyancy, such as active learning engagement and problem-solving [44,45]. Finally, considering emotional dimensions where students demonstrating buoyancy exhibit heightened levels of positive emotions such as joy, alongside decreased levels of negative emotions like boredom and anxiety [46,47]. The survey was administered in the participants’ native language to eliminate potential comprehension disparities arising from varying levels of English proficiency. The 32-item scale demonstrated a Cronbach’s alpha value of .96, indicating high reliability and suitability for further analysis in this study.

### Procedure

#### Data collection

Two questionnaires were administered in this study using a convenient sampling strategy. These questionnaires were created via an online survey platform (www.wjx.cn), with a link generated and disseminated to participants through Chinese social applications such as WeChat and QQ for data collection purposes. Before commencing the survey, approval of the research was granted by Human Research Ethics Committee of Hunan University. Additionally, prior to responding to the survey questions, students were initially verbally informed by their instructors that participation was anonymous and entirely voluntary. Subsequently, participants were required to read an informed consent statement with “Accept” and “Reject” buttons that appeared immediately after clicking on the survey link. This statement outlined the study’s purpose and procedures, provided assurance that participants could withdraw without penalty, and confirmed that their personal information would be protected. Participants could proceed to the formal survey by clicking the “Accept” button, while the “Reject” button allowed them to decline participation. The final data used in this study represents no objections to the survey. The data collection period for both questionnaires lasted one week. Sample 1 was collected from March 20^th^ to 24^th^, 2023, while Sample 2 was collected from April 2^nd^ to 12^th^, 2023.

#### Data analysis

In this study, the collected data were initially organized and screened, with problematic data, such as incomplete responses or those with identical options, excluded to ensure usability. The cleaned data were then recoded and uploaded to SPSS Statistics 26.0 and Mplus 8.3, where they were analyzed using a series of statistical procedures to address different research questions. In particular, the data from Sample 1 were analyzed using item analysis and EFA in SPSS to explore the probable factor structure of the construct of foreign language learning buoyancy. The data from Sample 2 were subjected to CFA and various types of validity tests, including construct validity, convergent validity, discriminant validity, in Mplus to assess the psychometric properties of the buoyancy construct. Finally, reliability tests, including internal consistency and split-half reliability, were conducted in SPSS.

## Results

### Academic buoyancy structure in EFL setting

Various metrics were used in the item analysis to demonstrate the relevance and reliability of each item within the buoyancy scale. These included item-total correlations (with a threshold above .40 deemed acceptable), the critical ratio method (requiring a significant *t*-value), and a homogeneity test (where the communality value should exceed .20 and factor loading values should be greater than .45) [48]. Table 2 lists the results of the item analysis. The overall data performed well on these critical metrics, with most values falling within acceptable ranges. However, there are some challenges in item 17. Three metrics failed to meet the thresholds, namely the values of the corrected item-total correlation is .39, the communality is .17, and the factor loading is .41. Therefore, the item will be temporarily removed from the subsequent analysis of this study.

**Table 2:** Results of the item analysis.

Item	Item-total correlation		*α*corrected	CR	Homogeneity test	
	*r*total	*r*corrected		*t-*value	communality	FL
**1**	.69**	.66	.96	-10.02**	.50	.70
**2**	.74**	.72	.96	-11.96**	.57	.75
**3**	.61**	.58	.96	-7.67**	.38	.62
**4**	.74**	.72	.96	-11.97**	.57	.75
**5**	.63**	.61	.96	-10.53**	.41	.64
**6**	.67**	.65	.96	-8.90**	.46	.68
**7**	.73**	.71	.96	-11.47**	.55	.74
**8**	.75**	.73	.96	-11.68**	.57	.76
**9**	.78**	.76	.96	-13.85**	.62	.79
**10**	.81**	.79	.96	-16.55**	.65	.81
**11**	.80**	.78	.96	-12.65**	.64	.80
**12**	.69**	.66	.96	-11.00**	.48	.69
**13**	.71**	.68	.96	-12.49**	.49	.70
**14**	.51**	.46	.96	-7.03**	.23	.48
**15**	.74**	.72	.96	-10.02**	.55	.74
**16**	.77**	.75	.96	-10.51**	.60	.77
**17**	.43**	.38	.96	-4.15**	.17	.41
**18**	.71**	.69	.96	-11.34**	.51	.71
**19**	.50**	.46	.96	-7.31**	.23	.48
**20**	.48**	.45	.96	-7.19**	.22	.47
**21**	.46**	.43	.96	-7.77**	.20	.45
**22**	.57**	.55	.96	-9.25**	.32	.57
**23**	.49**	.45	.96	-6.28**	.23	.48
**24**	.67**	.64	.96	-11.51**	.44	.66
**25**	.71**	.69	.96	-12.66**	.49	.70
**26**	.73**	.71	.96	-15.27**	.52	.72
**27**	.73**	.71	.96	-13.89**	.52	.72
**28**	.81**	.80	.96	-14.31**	.68	.82
**29**	.86**	.84	.96	-15.89**	.75	.87
**30**	.79**	.78	.96	-12.82**	.64	.80
**31**	.80**	.79	.96	-13.85**	.66	.81
**32**	.81**	.79	.96	-14.50**	.67	.82

Dimensionality reduction of the retained 31 items was performed using principal component analysis and varimax rotation in EFA. The value of the Kaiser-Meyer-Olkin (KMO) is .94 (acceptable value > .70), and Bartlett’s test of sphericity shows significant, with *χ2*(*n* = 209) = 3214. 41 (*p* < .001), indicating that the data are suitable for factor analysis. The minimum factor loading criterion was set at .40, and the benchmark for cross-factor loading values was not to exceed .20 [49]. Table 3 presents the results of the EFA, which confirmed three factors for the buoyancy scale. Ten items (numbered 5, 9, 10, 11, 13, 14, 19, 21, 28, and 29) were excluded, resulting in a 21-item scale for assessing learners’ buoyancy in foreign language learning.

**Table 3:** Results of the exploratory factor analysis.

Items	Factor1	Factor2	Factor3
**1**	.87		
**4**	.84		
**7**	.83		
**8**	.81		
**2**	.77		
**6**	.74		
**3**	.73		
**16**	.55		
**18**	.55		
**12**	.50		
**15**	.46		
**32**	.46		
**31**		.91	
**20**		.88	
**22**		.81	
**23**		.59	
**27**			.91
**24**			.89
**25**			.88
**26**			.85
**30**			.44

Note: *n* = 209; cumulative % of variance (rotated) = 66.57%.

In the results of EFA, most of the items under Factor 1 are related to describing traits such as persistence, efficacy, positive emotion (enjoyment), and engagement. For example, an item in this factor states, “I would see the situation as a challenge.” These characteristics are all associated with excellent quality and ability when students face adversity and challenges in foreign language learning. Additionally, these traits are linked to positive emotions and engagement when students learn a foreign language, even in the midst of difficult and tricky activities or tasks. Therefore, considering the commonality of these items, Factor 1 was characterized as “sustainability” with a positive attitude.

The items attributed to Factor 2 are mostly related to goals, such as “I hope to achieve good results in the foreign language exam,” aligning with previous research indicating that buoyant students can persevere in the face of adversity and setbacks to achieve a certain goal or result. Factor 2 was named “goal-orientedness.”

Finally, the items loaded onto Factor 3 are more closely associated with control, as exemplified by the statement “When I get a bad mark I’m often sure how I’m going to avoid that happening again”, which corresponds to previous research indicating that buoyant students possess a sense of being able to mange learning challenges. Factor 3 was designated as “controllability.”

Consequently, three dimensions, namely, “sustainability,” “goal-orientedness,” “controllability” that can be utilized to measure the academic buoyancy in foreign language learning were identified among Chinese EFL learners.

### Validation of the buoyancy structure

To assess the accuracy of the construct in reflecting the psychometric attributes of foreign language learners, CFA was employed to further refine and validate the three-dimensional construct and the 12-item measurement scale of academic buoyancy generated from the EFA. Five indicators were used to present the fit of the CFA model: 1) chi-square to degrees of freedom (*χ2/df*); 2) comparative fit index (CFI); (3) Tucker-Lewis Index (TLI); (4) standardized root of mean square residual (SRMR); (5) root-mean-square error of approximation (RMSEA).

**Fig 1:**
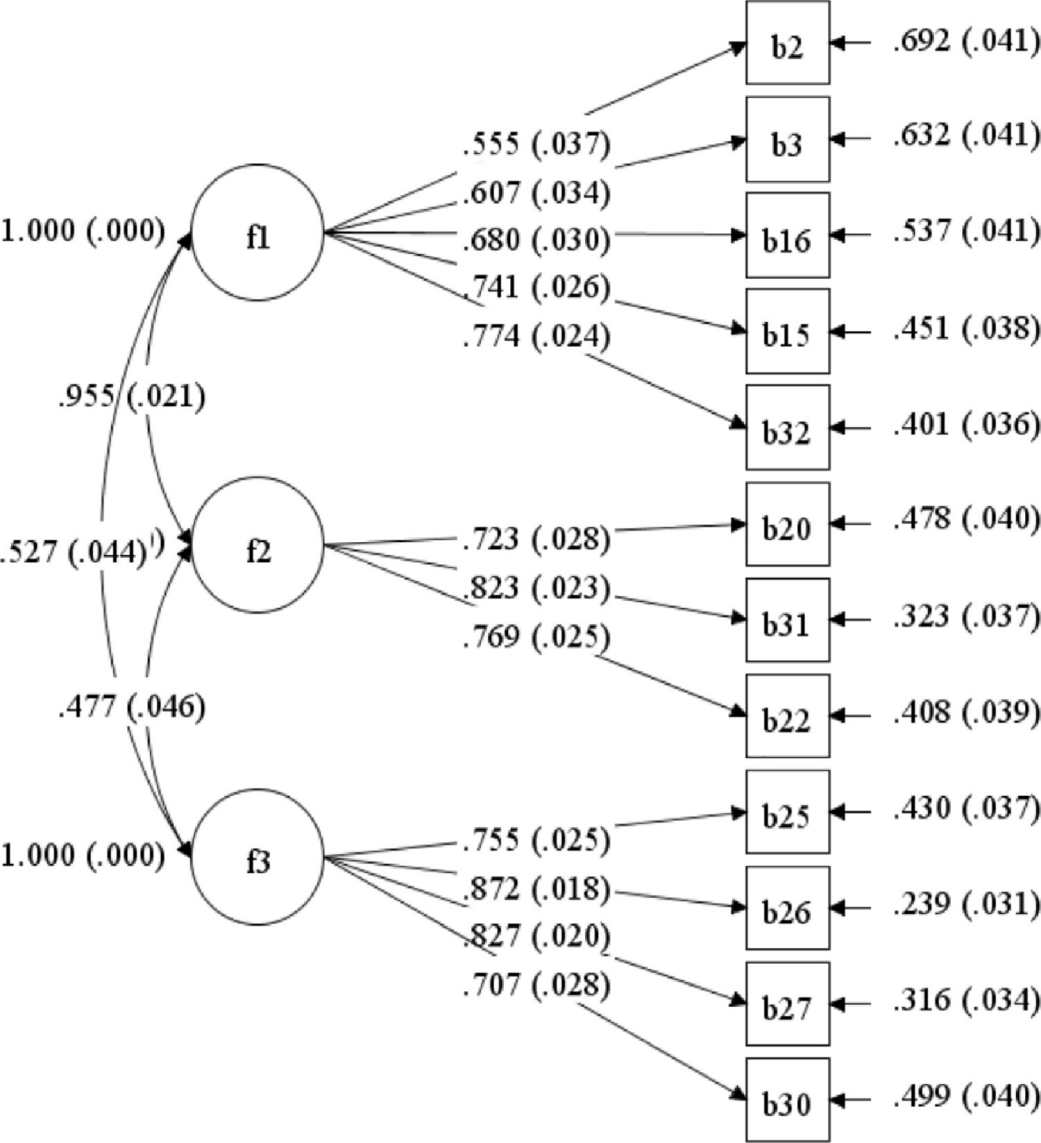
The final CFA model with 12 items for the buoyancy scale.

According to the acceptable benchmarks for model fit [50], the CFA model was a poor fit for the execution of the 21 items. All fit indicators were weak (CFI = .82, TLI = .80, RMSEA = .09, and SRMR = .08), except for the chi-square to degrees of freedom (*χ2/df* = 4.80), which fell within an acceptable range. This observation suggests that the model may not have been constructed optimally. Therefore, adjustments are necessary to improve its effectiveness. The data revealed that nine items had factor loading values less than .45. After removing these items and conducting a CFA on the remaining 12 items, a well-fitting model was obtained, with *χ2/df* = 3.22, CFI = .95, TLI = .94, RMSEA = .07, and SRMR = .04. Table 4 shows the results of the two executions of the CFA and Fig 1 demonstrates the final CFA model with 12 validate measurement items (see Appendix B for details of the items included in each factor).

**Table 4:** Results of the confirmatory factor analysis.

	*χ2*	*df*	*χ2/df*	CFI	TLI	RMSEA	SRMR
**Benchmarks**			< 5	> .90	> .90	< .08	< .08
**21-item CFA model**	892.36	186	4.80	.82	.80	.09	.08
**12-item CFA model**	164.08	51	3.22	.95	.94	.07	.04

The reliability of the final construct and measurements of the language learning buoyancy was determined by two kinds of reliability tests, as shown in Table 5. The reliability values of the scale for both tests performed well.

**Table 5:** Reliability for the 12-item buoyancy scale.

	Reliability test	split-half reliability test
**Factor 1: sustainability (5 items)**	.79	.73
**Factor 2: goal-orientedness (3 items)**	.77	.69
**Factor 3: controllability (4 items)**	.80	.79
**Overall scale (12 items)**	.86	.78

Furthermore, average variance extracted (AVE) analysis was ultimately used to verify whether the three factors (i.e., sustainability, goal-orientedness, and controllability) identified above could independently respond to the different dimensions of the buoyancy. Table 6 indicates the Pearson correlations between the factors and square root of AVE values (values on the diagonal) of the construct. It is required that if the square root of the AVE for each factor significantly exceeds its correlation with other factors, this implies discriminant validity in the model. The results showed poor discriminant validity between factor 1 (sustainability) and factor 2 (goal-orientedness).

**Table 6:** Pearson correlations and the square root of AVE.

	Factor 1	Factor 2	Factor 3
**Factor 1**	*0*.*68*		
**Factor 2**	0.96	*0*.*77*	
**Factor 3**	0.53	0.48	*0*.*79*

Note: Diagonal italic numbers are the square root of AVE values. Factor 1 = sustainability; Factor 2 = goal-orientedness; Factor 3 = controllability.

The three dimensions of the buoyancy structure were further analyzed with descriptive statistics. The results are shown in Table 7. Notably, the mean value for “goal-orientedness” stands the highest (*M* = 3.84), suggesting that English learners demonstrate a significant sense of purpose and direction in their academic pursuits, which serves as a primary capability in addressing difficulties and challenges in the process of foreign language learning. The following dimensions are “sustainability” (*M* = 3.61) and “controllability” (*M* = 3.38), with relatively small differences in mean values, suggesting a balanced distribution of abilities. Overall, Chinese EFL learners possess above-average buoyancy capabilities *(M* = 3.59), demonstrating that they have a strong ability to cope with challenges, and are able to maintain a positive attitude and effective learning strategies in complex learning environments.

**Table 7:** Results of the descriptive statistical analysis.

	*N*	*M*	*SD*	Kurtosis	Skewness
**Sustainability**	423	3.61	0.59	1.11	-0.11
**Goal-orientedness**	423	3.84	0.63	1.05	-0.45
**Controllability**	423	3.38	0.72	0.01	0.24
**Total**	423	3.59	0.54	1.24	0.12

## Discussion

This study explored the psychometric properties of the structure and measurement scale in assessing learners’ buoyancy reactions within the context of EFL learning. The results indicate that sustainability, goal-orientedness, and controllability are robust dimensions in the construction of foreign language learning buoyancy. The nature of these factors presents essential aspects that are pivotal for learners to navigate the challenges inherent in EFL learning environments and maintain a resilient and positive attitude towards their language learning endeavors.

The “sustainability” dimension, with its positive attitudinal attributes, implies that buoyancy in foreign language learning is characterized by persistence, efficacy, positive emotion (enjoyment), and engagement [18,27,33,34]. It represents the ability to stay afloat and maintain a positive and proactive attitude, even in the face of challenges and difficulties [7]. As evidenced by previous research, sustainability underscores the importance of endurance and persistence in the face of setbacks and obstacles encountered during the language learning process [51,52]. Learners who possess a high level of sustainability are more likely to persevere through difficulties, thereby enhancing their overall language learning outcomes [23].

“Goal-orientedness” means that buoyancy could be performed as having clear and related goals in foreign language learning. Learning goals can motivate and guide learning behaviors, helping students concentrate on specific tasks and knowledge, thereby improving learning efficiency [53]. Additionally, it enables students to measure their learning progress, allowing for adjustments in learning strategies as needed, providing students with both motivation and direction in their studies [42]. It resonates with the goal-setting theory proposed by Locke and Latham [53], which posits that setting clear and specific goals enhances motivation and performance. Research by Dörnyei and Ushioda supports the significance of goal-setting in language learning, highlighting its role in fostering learner autonomy and motivation [54].

The third dimension, “controllability,” focuses on a student’s sense of being able to control their attitude and thought toward setbacks and challenges in foreign language learning. For example, if students attribute an academic setback to something internal and controllable (e.g., not studying hard enough), then they are more likely to attempt to alter the situation (i.e., by studying harder next time) to avoid a repetition of the outcome, which indicates high buoyancy [28]. In contrast, if the cause of the academic setback is perceived as being external and/or uncontrollable (such as poor teaching), students may feel there is nothing they can do to change the outcome next time. Thus, they may not change their approach to foreign language learning in the face of another upcoming challenge which represent low buoyancy [36]. Overall, the recognition of controllability in buoyancy empowers learners to take ownership of their language acquisition journey and to make conscious efforts to improve their language skills, ultimately leading to greater proficiency and confidence in learning a foreign language.

Furthermore, the findings of the present research partially accord with Martin and Marsh’s “5C Model” of academic buoyancy, which includes control, confidence (high self-efficacy), coordination (high planning), composure (low anxiety), and commitment (high persistence) [6]. They both emphasize the structure properties of control. The subtle difference between them lies in that the contrast of anxiety is not indicated in present study, which tends to more emphasize positive emotion and attitude when facing adversities and challenges. There is a similar structure between the current study and the study by Jahedizadeh et al. [23], as both studies focus on the structure of sustainability in buoyancy. Unlike in previous studies, the structure of academic buoyancy among Chinese students exhibited more goal-related manifestation. This may be because Chinese university students often have a clear and utilitarian purpose for studying foreign languages.

Generally, the data in this study exhibited satisfactory reliability, and the model demonstrated good fit, along with correlation observed between the measurement scales and the three factors of the buoyancy structure. The results explained to some extent the psychometric properties of buoyancy among Chinese EFL learners. However, the discrimination between factors of “sustainability” and “goal-orientedness” was not significant. It is suggested that the main reason for this phenomenon may be the existence of essentially overlapping statements when summarizing and modifying the 32 items from nine previous scales for the items analyzed in this study. This is one of the significant reasons for the poor discriminant validity between these two factors. In subsequent studies, data collection could be enhanced through interviews to construct a pool of measurement items, thereby refining the scale’s development.

Among the three dimensions of foreign language learning buoyancy, students exhibit the highest performance in the dimension of goal-orientedness, followed by sustainability, with the lowest in controllability. It may be attributed to a combination of factors such as the clarity of learners’ motivation, the richness of the learning environment and resources, as well as in terms of learning autonomy, emotional management and coping strategies.

College students often have clear learning goals and motivations in English learning, such as passing CET-4 or CET-6 exams, studying abroad, or seeking employment. Meanwhile, universities provide relatively abundant learning resources and environments, such as libraries and e-learning platforms, which aid students in achieving their learning objectives. Secondly, college students have gradually formed their own learning habits, attitudes as well as learning strategies and methods in the process of English learning. While they are able to maintain a certain degree of learning sustainability, this may be challenged by other factors or the immaturity and imperfection of their strategies and methods. Lower controllability may be due to students’ lack of sufficient autonomy and over-reliance on classroom and teachers. In the face of setbacks and failures, some students may lack effective emotional regulation and coping strategies, leading to frustration or abandonment in the face of difficulties.

Therefore, the findings of this study carry several pedagogical implications. By concentrating on these three key dimensions, educators can implement strategies to strengthen students’ academic buoyancy in foreign language learning. Concretely, with regard to sustainability, English instruction should prioritize the cultivation of positive attitudes toward foreign language learning and the development of emotional regulation skills to help students navigate academic challenges [47]. This approach enables students to persist in their foreign language studies despite facing various obstacles.

Secondly, in terms of goal-orientedness, teachers could assist students in setting realistic and attainable goals for their foreign language learning, tailored to students’ individual differences. Moreover, educators should guide students in deconstructing these long-term goals into a series of specific, actionable steps [53]. As students systematically complete incremental tasks, they cultivate a sense of accomplishment, which subsequently enhances their motivation to engage in the learning process.

Lastly, concerning controllability, educators should actively encourage students to express themselves, ask questions, and participate in class discussions. This practice not only develops their oral communication skills but also boosts their self-confidence and sense of control in the learning process [12,14]. Furthermore, timely and constructive feedback is crucial, as it helps students identify their strengths and areas for growth, reinforcing their sense of progress. By prioritizing the learning process over final outcomes, teachers can celebrate effort and improvement, promoting a more positive attitude toward language acquisition.

To enhance learners’ performance across various dimensions of the academic buoyancy, corresponding measures should be taken, such as clarifying learning goals, promoting the development of autonomous learning skills, strengthening emotional management and coping strategies, and providing a more supportive and resource-rich learning environment. By comprehensively implementing strategies focused on these three dimensions, educators can significantly enhance students’ academic buoyancy, facilitating their steady progress in foreign language learning.

## Conclusion

The present study has yielded two important findings. Firstly, the structure of academic buoyancy in foreign language learning was explored, which with characteristics of sustainability, goal-orientedness, and controllability. It can explain students’ ability to effectively cope with setbacks and challenges in the EFL context. Secondly, corresponding scales to measure buoyancy were confirmed, comprising 12 items. The findings of this study expand the scope of buoyancy research to some extent, providing new empirical support for existing academic buoyancy structures. It offers valuable insights for researchers and educators seeking a deeper understanding of buoyancy.

Inevitably, there are some potential limitations of this study. The data presented in this study are self-reported. This raises concerns regarding validity, reporting biases, the accuracy of recall, and impression management. Furthermore, the selected items in the investigations encompassed psychological factors like self-efficacy, control, learning goals, and school-related engagement factors. However, family and peer factors, which are equally important to learners’ learning experience, were not taken into account in the current study. Although multilevel modeling in Martin and Marsh [6] found that the majority of the variance in academic buoyancy was explained at the student level, a subsequent study is necessary to include family and peer factors (such as family support, positive relationships with pro-social adults, peer commitment to education, and authoritative and caring parenting, etc.). More importantly, since there are fewer studies related to buoyancy centered on foreign language learning. The measurement items in this study are a preliminary exploration of buoyancy by integrating resources from past empirical studies.

Future research could employ a mixed-methods approach, combining qualitative and quantitative techniques, and incorporating additional relevant factors related to family dynamics and peer influences to more comprehensively establish and enrich the conceptualization of foreign language learning buoyancy.

## Supporting information

S1 File Item analysis and EFA is the S1 File Title. This is the data collected during the first phase, which is used for project analysis and exploratory factor analysis.
(XLSX)

S2 File CFA is the S2 File Title. This is the data collected during the second phase, which is used for confirmatory factor analysis.
(XLSX)

S1 Appendix (DOCX)
