# Supplementary material for: Overlap or breakthrough? exploration of the academic buoyancy structure in Chinese EFL learners
Source: PLoS One. 2025 Jan 31;20(1):e0318347. doi: 10.1371/journal.pone.0318347 (PMC11785288; doi:10.1371/journal.pone.0318347)
Supplement: S1 Appendix — (DOCX) [file pone.0318347.s003.docx]

**Appendix**

Appendix A. The 32-item foreign language learning buoyancy scale《外语学习复原力量表》

| No. | Items |
| --- | --- |
| 1 | I would work harder  我会更努力地学习外语 |
| 2 | I would not change my long-term goals and ambitions  我不会轻易改变自己的长期外语学习目标 |
| 3 | I would see the situation as a challenge  我将眼前外语学习的困境看成是挑战 |
| 4 | I would see the situation as temporary  我会将困境看成是暂时的现象 |
| 5 | I would do my best to stop thinking negative thoughts  我会尽力停止负面思考 |
| 6 | I manage to carry out most of the school work that is given to me.  我会设法完成大部分家庭作业 |
| 7 | I am able to achieve success in learning on my own.  我能通过自学达到外语学习的目标 |
| 8 | When faced with severe problems in learning, I am able to solve them.  我能够解决学习中遇到的困难 |
| 9 | I am only physically in the classroom, while my mind is wandering outside the English class.  外语课上我人在教室，思想却在神游 |
| 10 | I get restless and can’t wait for the English class to end.  外语课上我经常有听不下去的感觉 |
| 11 | I enjoy it.  我享受外语学习 |
| 12 | In class, I feel proud of my accomplishments.  在班里，我为自己的外语成绩感到自豪 |
| 13 | It embarrasses me to volunteer answers in my FL class  我在外语课堂上发言会感到尴尬 |
| 14 | Even if I am well prepared for FL class, I feel anxious about it  即便我准备好了，在外语课上也会感到紧张 |
| 15 | I am able to recognize the causes of the learning problems I face.  我能够找出在学习中遇到困难的原因 |
| 16 | I am able to introspect myself when facing problems in learning.  当学习中遇到困难时，我能够自省 |
| 17 | I have difficulty in finding solutions to the problems I face when studying.  当学习中遇到困难时，我很难找到解决的办法 |
| 18 | I am able to think positively when facing a problem in learning.  当学习中遇到困难时，我会往积极的方向思考 |
| 19 | Other people are responsible for the failures I face at school.  当学习中遇到挫败时，我会责怪其他人 |
| 20 | I hope to achieve good results in the foreign language exam.  我希望在外语考试中能取得好成绩 |
| 21 | I have hope to be able to finish school on time.  我希望能够听懂外语对话和演讲 |
| 22 | I have a clear plan of what I will bend after graduating from school.  我希望能够阅读和理解外语的各种文章、报纸、小说等 |
| 23 | My language learning goal is to obtain language proficiency certificates through relevant exams, such as the CET-4 and CET-6.  我的外语学习目标是通过相关考试获得外语水平证书，如四六级等 |
| 24 | When I get a good mark I’m often sure how I am giong to get that mark again  当我得到一个不错的分数之后，能确定如何再得到这个分数 |
| 25 | When I don’t do so well at school I'm often sure how to avoid that happening again  当我在学校表现不好时，我知道如何避免其再发生 |
| 26 | When I get a bad mark I’m often sure how I'm going to avoid that happening again.  当我分数不好时，我知道如何避免再发生 |
| 27 | I often feel sure about how I can avoid doing poorly in foreign language learning.  我知道怎么避免在外语学习时的不良表现 |
| 28 | I listen carefully in class.  我上课认真听讲 |
| 29 | I pay attention in class.  我上课注意力集中 |
| 30 | The first time my teacher talks about a new topic, I listen very carefully.  当老师提出一个新话题时，我会仔细听 |
| 31 | I hope to improve my abilities through learning.  我希望通过学习提升自己的能力 |
| 32 | I work hard when we start something new in class  当学习新知识时，我会很努力 |

Appendix B. The 12-item foreign language learning buoyancy scale《外语学习复原力量表》

| No. | Items |
| --- | --- |
| Factor 1: Sustainability | |
| 2 | I would not change my long-term goals and ambitions  我不会轻易改变自己的长期外语学习目标 |
| 3 | I would see the situation as a challenge  我将眼前外语学习的困境看成是挑战 |
| 15 | I am able to recognize the causes of the learning problems I face.  我能够找出在学习中遇到困难的原因 |
| 16 | I am able to introspect myself when facing problems in learning.  当学习中遇到困难时，我能够自省 |
| 32 | I work hard when we start something new in class  当学习新知识时，我会很努力 |
| Factor 2: Goal-orientedness | |
| 20 | I hope to achieve good results in the foreign language exam.  我希望在外语考试中能取得好成绩 |
| 22 | I have a clear plan of what I will bend after graduating from school.  我希望能够阅读和理解外语的各种文章、报纸、小说等 |
| 31 | I hope to improve my abilities through learning.  我希望通过学习提升自己的能力 |
| Factor 3: Controllability | |
| 25 | When I don’t do so well at school I’m often sure how to avoid that happening again  当我在学校表现不好时，我知道如何避免其再发生 |
| 26 | When I get a bad mark I’m often sure how I’m going to avoid that happening again.  当我分数不好时，我知道如何避免再发生 |
| 27 | I often feel sure about how I can avoid doing poorly in foreign language learning.  我知道怎么避免在外语学习时的不良表现 |
| 30 | The first time my teacher talks about a new topic, I listen very carefully.  当老师提出一个新话题时，我会仔细听 |
